# A Whole Genome Sequencing-Based Approach to Track down Genomic Variants in Itraconazole-Resistant Species of *Aspergillus* from Iran

**DOI:** 10.3390/jof8101091

**Published:** 2022-10-17

**Authors:** Sanaz Nargesi, Reza Valadan, Mahdi Abastabar, Saeed Kaboli, Jose Thekkiniath, Mohammad Taghi Hedayati

**Affiliations:** 1 Department of Medical Mycology, Faculty of Medicine, Mazandaran University of Medical Sciences, Sari 4815733971, Iran; 2 Invasive Fungi Research Center, Communicable Diseases Institute, Mazandaran University of Medical Sciences, Sari 4815733971, Iran; 3 Department of Immunology/Molecular and Cell Biology Research Center (MCBRC), Mazandaran University of Medical Sciences, Sari 48175-1665, Iran; 4 Department of Medical Biotechnology, School of Medicine, Zanjan University of Medical Sciences, Zanjan 4513956111, Iran; 5 Cancer Gene Therapy Research Center, Zanjan University of Medical Sciences, Zanjan 4513956111, Iran; 6 Fuller Laboratories, 1312 East Valencia Drive, Fullerton, CA 92831, USA

**Keywords:** *Aspergillus* spp., itraconazole resistance, *cyp51* genes, whole genome sequencing

## Abstract

The antifungal resistance in non-fumigatus *Aspergillus* spp., as well as *Aspergillus fumigatus*, poses a major therapeutic challenge which affects the entire healthcare community. Mutation occurrence of *cyp*51 gene paralogs is the major cause of azole resistance in *Aspergillus* spp. To obtain a full map of genomic changes, an accurate scan of the entire length of the *Aspergillus* genome is necessary. In this study, using whole genome sequencing (WGS) technique, we evaluated the mutation in *cyp51A*, *cyp51B*, *Cdr1B, AtrR, Hmg1, HapE* and *FfmA* genes in different clinical isolates of *Aspergillus fumigatus*, *Aspergillus niger*, *Aspergillus tubingensis*, *Aspergillus welwitschiae* and *Aspergillus terreus* which responded to minimum inhibitory concentrations of itraconazole above 16 µg mL^−1^. We found different nonsynonymous mutations in the *cyp51A*, *cyp51B*, *Cdr1B, AtrR, Hmg1, HapE* and *FfmA* gene loci. According to our findings, *Aspergillus* species isolated from different parts of the world may represent different pattern of resistance mechanisms which may be revealed by WGS.

## 1. Introduction

The mycoses caused by *Aspergillus* spp. include a wide variety of pathophysiological disorders, ranging from allergic manifestations to systemic infections [1]. Among the invasive fungal diseases (IFD), invasive aspergillosis (IA) is one of the most severe clinical entities, with mortality rates up to 70% in more than 300,000 cases estimated worldwide [2]. Although several studies have been focused on the most common species of *Aspergillus* including *A. fumigatus* and *A. flavus*, using molecular taxonomic tools, cryptic/rare *Aspergillus* species have recently been identified [3]. Hence, there is a growing awareness about the clinical relevance of *Aspergillus* cryptic species in the development of IA [4,5]. According to clinical observations, the epidemiological distribution of cryptic *Aspergillus* species are associated with invasive infections and often falls into the sections *Fumigati*, *Flavi*, *Nigri*, and *Terrei* [6]. Azole derivatives are regarded as the first-line options for the prophylactic treatment of IA [7]. Among azole antifungals, itraconazole (ITC) is widely used in medicine and veterinary treatment. This antifungal agent was considered as the treatment of choice for chronic pulmonary aspergillosis [8], and have been proposed as an alternative treatment for allergic bronchopulmonary aspergillosis and the management of patients with severe asthma [9] An unsettled dilemma in managing aspergillosis crisis is the emergence of azole-resistance in *Aspergillus* spp., a phenomenon that stems from intricate mechanisms [10]. Due to the global rampancy of *A. fumigatus*, major studies have been steered to scrutinize the mechanisms and understand the roots of resistance against triazoles derivatives including ITC in environmental and clinical isolates [11,12,13]. With trailing the function of genes, it is inferred that occurrence of point mutations in *cyp*51 gene paralogs (*cyp51A* and *cyp51B*) in *A. fumigatus*, *A. niger* and *A. terreus* are the most common genomic changes leading to azole resistance [14]. In response to triazoles acting on the locus of *cyp51A* and *cyp51B* genes, resulting mutation manifests as a non-synonymous substitution that alters the sequence of encoded proteins and thus resistant mutants are aroused [15,16]. However, mutation in non-*cyp*51 gene including ATP-binding cassette transporters (*Cdr1B* or *abcG1*)*, AtrR, Hmg1, HapE* and *FfmA* gene loci have been recently reported [17,18,19,20,21]. In recent years, whole genome sequencing (WGS) and bioinformatics methodologies have gained traction in the diagnostic field as potent appliances for detecting antifungal resistance in medical mycology [22]. Using WGS with a high number of clinical and environmental isolates of *A. fumigatus*, Rhodes et al. [23] showed that a strong genetic structuring into different clades with little interclade recombination, high confidence documents to show that transmission of resistant isolates occurs from the environment to the infected patients. The selective sweeps across multiple regions indicate a polygenic basis to traits in some genetic backgrounds, and the genes related to the azole resistance with previously unknown resistance mechanisms. Since WGS is a high-throughput technology that reads out the whole genomic content, it provides high-resolution images of single nucleotide polymorphisms (SNPs) at different points in the DNA of the target microorganism, which allows for wider genomic analysis as compared to primer design -based techniques [24]. In light of lack of information on WGS related to azole-resistant *Aspergillus* spp. and the importance of clinically relevant and cryptic species of *Aspergillus*, we evaluated the function of WGS to detect the genomic variations of *cyp51A*, *cyp51B*, *Cdr1B, AtrR, Hmg1, HapE* and *FfmA* genes in ITC-resistant isolates of *A. fumigatus* (section *Fumigati*), *A. terreus* (section *Terrei*), *A. niger*, *A. tubingensis*, and *A. welwitschia* (section *Nigri*).

## 2. Materials and Methods

### 2.1. Clinical Isolates

A total of seven identified (using molecular techniques by sequencing of the β-tubulin-encoding gene (*benA*)) *Aspergillus* isolates including *A. fumigatus* (AF), *A. niger* (AN) and *A. welwitschiae* (AW), and *A. tubingensis* (AT1 and AT2) and *A. terreus* (AR1 and AR2) that responded to minimum inhibitory concentrations (MICs) of ITC above 16 µg mL^−1^ were included in the study. Table 1 shows the clinical source and accession number of isolates.

### 2.2. Molecular Identification

DNA was extracted from fungal colonies grown for 5–7 days on malt extract agar (Difco, Detroit, MI, USA) using a previously described method [25]. Polymerase chain reaction (PCR) for partial amplification of the *benA* was performed using forward and reverse primers Bt2a (5′-GGTAACCAAATCGGTGCTGCTTTC-3′) and Bt2b (5′-ACCCTCAGTGTAGTGACCCTTGGC-3′), respectively [26]. PCR products were analyzed by gel electrophoresis using 1.5% agarose gels, and the amplified *benA* fragments were sequenced using the same primers with the ABI3730xL Genetic Analyzer platform (Applied Biosystems, Waltham, MA, USA). All sequences were subjected to nucleotide BLAST search of the NCBI database and the molecular database of the WI-KNAW Fungal Biodiversity Center (Utrecht, The Netherlands), and isolates were identified up to the species level.

### 2.3. Antifungal Susceptibility Testing

Antifungal susceptibility test (AFST) was fulfilled as a 2× microdilution broth method based on the Clinical and Laboratory standards Institute (CLSI)-M38-A3 instructions [27]. The azole antifungals evaluated in this study were ITC, voriconazole (VOR), posaconazole (POS), and isavuconazole (ISV) that purchased from Merck Sharp and Dohme BV, Haarlem, The Netherlands. To test antifungal susceptibility, a suspension of each isolate was prepared in RPMI 1640 broth medium (absorbance reading, A_530_ of 0.09 to 0.13). Five azole derivatives mentioned above were also diluted in dimethyl sulfoxide (DMSO). *Aspergillus* suspensions in 96-well plates were exposed to azoles by 2× dilution (ranging from 0.016 to 16 μg mL^−1^) and incubated at 35 °C for 48 h. *Candida parapsilosis* (ATCC 22019) and *Pichia kudriavzevii* (ATCC 6258) were used as quality control strains. The MIC of the isolates was evaluated and after comparing with defined ECV values in CLSI supplement M59, non-wild type (NWT) isolates were identified.

### 2.4. Workflow of Whole Genome Sequencing (WGS)

DNA extraction was performed on each isolate after a three-day culture with the DNA extraction kit (SimBiolab, Mashhad, Iran). Genomic DNAs were quantified and assessed qualitatively using gel electrophoresis and NanoDrop spectrophotometer. The extracted gDNAs were transferred to Novogene (Tianjin, China) for genomic sequencing using GenTegra-DNA tubes (USA), which protect against degradation. Prior to starting the sequencing process, an RNAase treatment step was also considered to improve gDNAs purity. The DNAs were subjected to a 5400 fragment analyzer system, designed to meet the needs of ensuring their quantity, integrity and purity, as the preliminary assaying types. The libraries were prepared at Novogene Bioinformatics Technology Co., Ltd. (Beijing, China) using TruSeq^®^ DNA PCR-Free Sample Preparation Kit (Illumina, San Diego, CA, USA) according to manufacturer’s recommendation. In brief the genomic DNA was randomly sheared into short fragments. Then, the obtained fragments were end repaired, A-tailed and further ligated with Illumina adapter. The fragments with adapters were PCR amplified, size selected, and purified. The quality of the prepared libraries was assessed using Qubit and real-time PCR. At that time, a library was prepared according to each of the targeted isolates. After going through all these steps, all DNA samples were sequenced using paired-end 150 based pair sequencing. As part of our whole-genome sequencing project, we used an Illumina NovaSeq 6000 system, and the sequencing depth for the isolates was 100×. To conclude, data generated by Illumina platform were processed via CLC genomics workbench 21 software (QIAGEN GmbH, Hilden, Germany) according to following steps:

i. The forward and reverse sequencing data were imported into the software using Illumina High-Throughput Sequencing import option (Illumina Pipeline 1.8) in which the data were paired-ended, de-multiplexed and trimmed. The failed reads were also discarded.
ii. The quality of the sequencing data was assessed using quality control of the software which include a complete list of lengths and quality distribution, GC content, ambiguous base-content, nucleotide contributions, sequence duplication levels and the sequence of the duplicated reads.
iii. The reference sequence of each species was downloaded from NCBI (Table 2) and used as a reference for data mapping and resequencing using the Map Reads to Reference option of the software, then the quality of the mappings was assessed using QC for Read mapping and Genome Coverage Analysis option of the software.
iv. The variant calling of the assembled sequencing data was performed using Basic Variant Detection option of the software, then each variant was also annotated manually of the target genes for confirmation.
v. Finally, the functional consequences of the variations was determined using Amino Acid Change option of the software by comparison of the variant tracks of the assembled sequence with the references.

**Figure 1:**
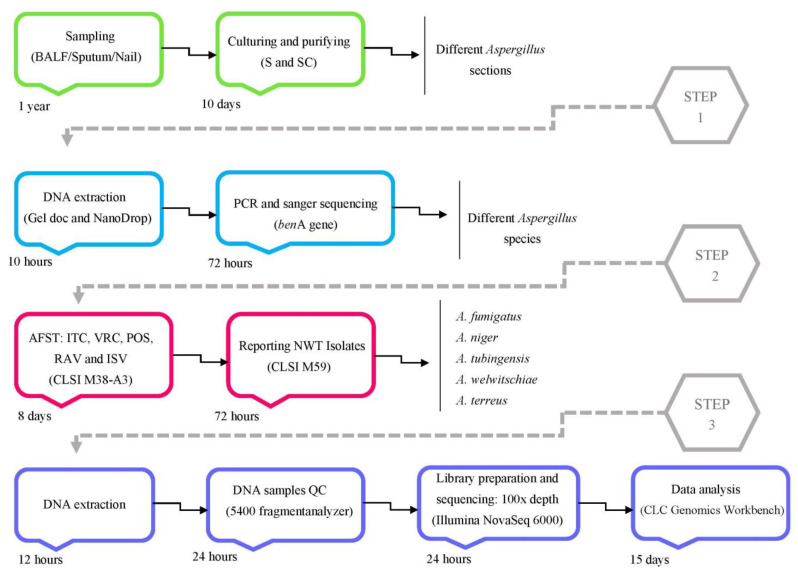
The steps of project implementation, from sample collection to WGS. The estimated time to complete each of the key stages of the project indicates that after the sampling phase, which spans a period of one year, within two months, it is possible to analyze azole-resistant *Aspergillus* spp. through whole genome sequencing to identify all SNPs in the entire genomic content.

All DNA sequence data were uploaded to NCBI SRA-submission portal (https://submit.ncbi.nlm.nih.gov/subs/sra/ (accessed on 20 Novamber 2021)). In Figure 1, the steps from the research are depicted.

### 2.5. Homology Modeling and Mutation Mappings

The primary amino acid sequence for CYP51A of ***A. fumigatus*** (XP_752137.1), ***A.welwitschiae*** (XP_026629604.1), ***Aspergillus niger*** (XP_001394224.1), ***Aspergillus tubingensis*** (XP_035357535.1) and ***Aspergillus terreus*** (XP_001215095.1) were used to find appropriate template for homology modeling of the 3D structure of each protein using ICM-Pro software (Molsoft L.L.C., San Diego, CA, USA). Each template was imported into the software, refined and minimized then used for homology modeling as described previously [28].

## 3. Results

### 3.1. Determination of MIC 

**Figure 2:**
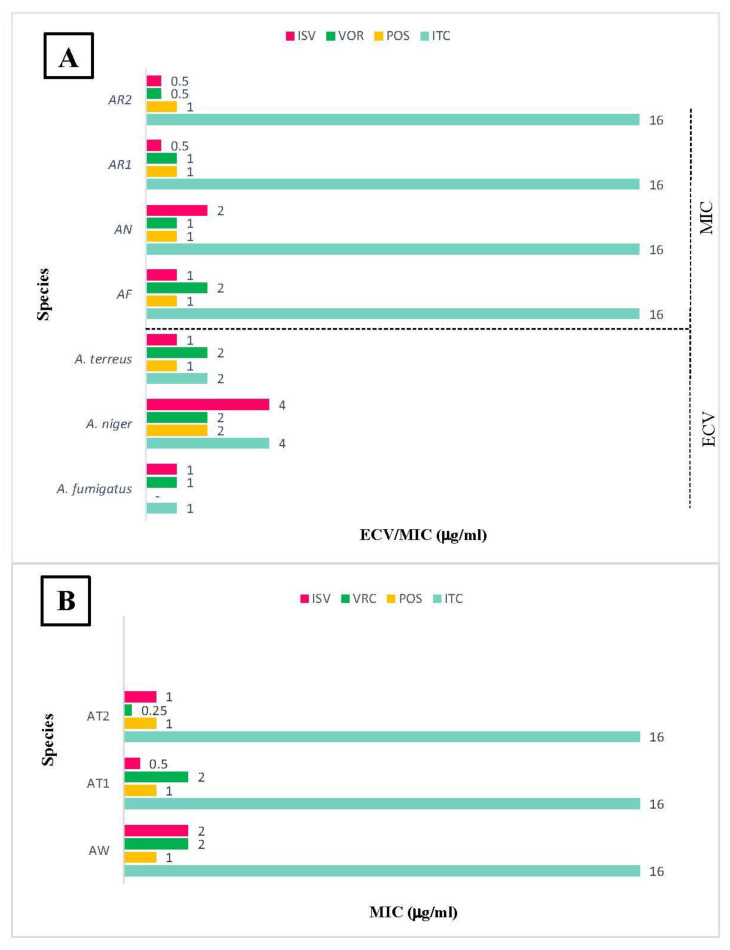
(**A**) The distribution of minimum inhibitory concentrations (MICs) of different *Aspergillus* isolates against tested triazoles compared to epidemilogical Cutoff values (ECVs). (**B**) The MICs of different *Aspergillus* isolates without defined ECV against tested triazoles.

The MIC values for seven isolates tested with ITC, VOR, POS, and ISV are provided in chart 1. Due to the presence of an ECV definition in CLSI M59 supplement for *A. fumigatus*, *A. niger*, and *A. terreus*, the MIC profiles of the four isolates AF, AN, AR1, and AR2 were comparable. AF, AN, AT1 and AT2 response to ITC with MIC > 16, POS = 1, VOR = 2, 1, 1 and 0.5, ISV = 1, 2, 0.5 and 0.5, respectively. The four isolates with MIC > 16 to ITC were therefore identified as NWT isolates (Figure 2A). For the three isolates AW, AT1 and AT2, there was no standard ECV to comparison. AW, AT1 and AT2 isolates showed MIC > 16 to ITC, MIC = 1 to POS, MIC = 2, 2 and 0.25 to VOR and MIC = 2, 0.5 and 1 to ISV, respectively (Figure 2B).

The detailed information on the isolates is also accesible in our recent published paper [3].

### 3.2. DNA Quality Control (QC) Report

During quality control, all seven isolates passed the criteria for integrity, concentration, and purity. DNA concentrations were between 50 and 75 ng/μL, and integrity tests yielded satisfactory upshots.

### 3.3. Homology Modeling and Mutation Mappings

Conformational structure of A. welwitschiae was not shown as the V16L mutation is located on the signal peptide of CYP51A.

**Figure 3:**
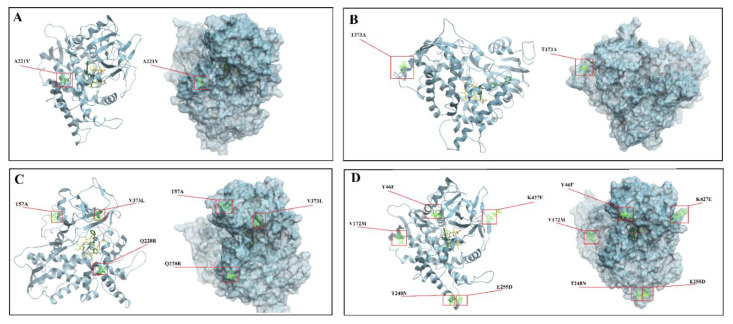
Homology modeling of CYP51A of the selected Aspergillus species and mutation mapping on the structures. Each structure was shown in two different presentations. (**A**) (*A. terreus*), (**B**) (*A. tubingensis*), (**C**) (*A. niger*) (**D**) (*A. fumigatus*).

In the current study, different mutations were identified and mapped on the structure of the modeled CYP51A. As illustrated in Figure 3, some mutations were located near the channel entry especially in *A. Fumigatus* (Figure 3D) but far from the active site of the proteins. This could provide a potential mechanism for azole resistance by limiting the access of inhibitors to the active site.

### 3.4. Bioinformatics Analysis

Upon alignment of the sequences of *cyp51A*, *cyp51B*, *Cdr1B*, *AtrR*, *Hmg1*, *HapE* and *FfmA* as target genes confines of the seven isolates with the reference sequences deposited in NCBI, genomic variations were detected at different positions, which are depicted in Table 2. The intron sequences have been removed in the raw data processing process. The SNP positions in Table 2 are based on DNA coding sequences (CDS) that corresponds to the sequence of amino acids in a protein. Four nonsynonymous mutations have been identified in *cyp51A* sequence (T137A (F/Y), A514G (M/V), A743T (N/T) and C765G (D/E)) of *A. niger* isolate. Although the occurrence of the other four SNPs did not start off amino acid changes (G267A, A1047G, G1279A and T1362C). *A. niger* isolate contained three nonsynonymous substitutions (A169G (T/A), A683G (Q/R) and G1147C (V/L)) and eleven silent substitutions across the *cyp51A* gene. In *cyp51B* gene, variation at points A800T (N/I) and A1184C (N/T) also altered the protein sequence. A solitary A. welwitschiae (AW) isolate was consorted with one and three replacement mutations in *cyp51A* (G46C (V/L)) and *cyp51B* genes (A440G (E/G), T525A (N/K), and A2785G (I/V)), respectively. Two isolates of *A. tubingensis* with AT1 and AT2 codes revealed dissimilar genomic changes in the *cyp51A* and *cyp51B* genes. The former showed only one nonsynonymous mutation in the *cyp51A* target (C166T (L/F)) and the latter showed the same pattern as the reference genome in the *cyp51A* target. The amino acid sequences of isolates AT1 and AT2 were unaffected by point mutations in the *cyp51A* and *cyp51B* genetic codes. *A. terreus* isolates also gave different results. For AR1, replacement mutations at C662T (A/V) of the *cyp51A* gene, and at T1166G (T/S) of the *cyp51B* gene were unmask. In the second isolate of *A. terreus*, AR2, a silent mutation was spotted in the *cyp51A* gene, while no meaningful variance was stood up along the *cyp51B* gene as compared to reference sequence. It has been found that all seven AN, AF, AW, AT1, AT2, AR1 and AR2 isolates ultimately showed resistance to ITC following occurrence of substitution mutations at different loci of *cyp51A* and *cyp51B* and as target genes under study (Table 2). In non-cyp51 gene loci, nonsynonymous mutations have been identified in different isolates as follows:

*A. welwitschiae* in *Cdr1B* (one mutation), *Hmg1* (five mutations) and *HapE* (one mutation).

*A. fumigatus* in *AtrR* and *Hmg1* (two mutations, each).

*A. niger* in *Cdr1B* (four mutations), *AtrR* (two mutations) and *Hmg1* (one mutation).

Isolate AT1 of *A. tubingensis* in *Cdr1B* (tow mutations), *HapE* (one mutation) and *FfmA* (two mutations). Isolate AT2 in *AtrR* and *FfmA* (two mutations, each).

Isolate AR1 of *A. terreus* showed one mutation in *HapE* locus and AR2 in *Cdr1B* (one mutation), and *AtrR* (tow mutations) (Table 2).

Our data is associated with an NCBI BioProject accession, PRJNA845900.

**Table 2:** The list of nonsynonymous substitutions along different gene loci in study *Aspergillus* isolates.

Species	BioSample Accessions	*Cyp51A*	*Cyp51B*	*Cdr1B*	*AtrR*	*Hmg1*	*HapE*	*FfmA*	Reference Accession Number
AW	SAMN28864578	G46C (V/L)	A561G (E/GT646A (N/K) A4438G (I/V)	C4755T (H/Y)	-	G219A (G/N) G498A (G/E) T1342C (V/A) G1524C (V/L) C1828G (T/S)	A133C (K/T)	-	GeneID:38137106 GeneID:38137970 GeneID:38143887 GeneID:38132489 GeneID:38143658 GeneID:38145110 GeneID:38137721
AF	SAMN28864579	A137T (Y/F) G514A (V/M) C743A (T/N) G765C (E/D) A1279G (K/E)	-	-	G2092C (H/D) C2272T (P/S)	T697C (S/P) T1803C (Y/H)	-	-	GeneID:3509526 GeneID:3506370 GeneID:3509814 GeneID:3506650 GeneID:3506671 GeneID:3505192 GeneID:3512958
AN	SAMN28864580	A267G (T/A) A838G (Q/R) G1302C (V/L)	A1072T (N/I) A11456C (N/T)	G1905T (A/S) A1996G (D/G) C2921A (D/E) CT4987-8TC (Y/H)	T2417C (F/S) G2794A (A/T)	T1605G (D/E)	-	-	GeneID:4984452 GeneID:4986453 GeneID:4977469 GeneID:4981973 GeneID:4982097 GeneID:37104718 GeneID:4979089
AT1	SAMN28864581	C383T (L/F) A1390G (T/A)	-	G1069A (V/I) T4554G (S/A)	-	-	C756G (A/G)	T1293C(S/P) T1468C(F/S)	GeneID:56006014 GeneID:56008603 GeneID:56003599 GeneID:56007043 GeneID:56006955 GeneID:56001387 GeneID:56005349
AT2	SAMN28864582	-	-	-	C2687A (T/N) A2767G (T/A)	-	-	T1293C(S/P) T1468C(F/S)	GeneID:56006014 GeneID:56008603 GeneID:56003599 GeneID:56007043 GeneID:56006955 GeneID:56001387 GeneID:56005349
AR1	SAMN28864583	C706T (A/V)	C1280G (T/S)	-	-	-	T1103C (F/S)	-	GeneID:4321797 GeneID:4317693 GeneID:4319285 GeneID:4318923 GeneID:4354193 GeneID:4321255 GeneID:4315909
AR2	SAMN28864584	-	-	A3279T (E/D)	A70C (N/H) A2735C (Q/H)	-	-	-	GeneID:4321797 GeneID:4317693 GeneID:4319285 GeneID:4318923 GeneID:4354193 GeneID:4321255 GeneID:4315909

## 4. Discussion

Among susceptible patients with impaired immune functions for a variety of reasons, such as solid organ transplant recipients and hematopoietic stem cell transplant recipients, *A. fumigatus*, *A. niger*, *A. terreus*, and *A. flavus* are the most common isolated species of IA, but the role of cryptic species should not be overlooked [29]. Species that appear to be cryptic might be neglected because of misidentification and therefore, it would be difficult to assess their epidemiological distribution pattern and antifungal susceptibility profile [5]. Antifungal susceptibility pattern, and rate and mechanism of resistance in ITC as a main agent in management and prophylaxis of aspergillosis against different species of *Aspergillus* have been analyzed in different countries [30,31,32,33,34,35]. In vitro activity of azoles determined by CLSI method against different opportunistic filamentous fungal pathogens including *Aspergillus* species from the Asia and Western Pacific Region, the data from the SENTRY Antifungal Surveillance Program (2011–2019) showed the ITC MIC_50_/_90_ as 0.5/1 mg L^−1^ for *A. fumigatus*, *A. flavus* and *A. terreus* and 1/2 mg L^−1^ for *A. niger*. The overall frequency of NWT strains (MICs above the ECVs) of *A. fumigatus*, *A. flavus*, *A. terreus* and *A. niger* were 2.1%, 0, 0 and 2.2% for ITC, respectively [30]. The similar MIC_50_/_90_ results for *A. fumigatus*, *Aspergillus* section *Flavi*, *Terri* and *Nigri* were reported in another large-scale study which analyzed 1775 isolates of *Aspergillus* spp. collected from Europe, North America, Latin America, and Asia-Pacific region from 2010–2017 [31]. They also reported an overall frequency of NWT strains of *A. fumigatus* as 0.9% for ITC. In addition, Europe displayed the highest NWT rates for ITC (1.7%) when compared to other regions (0.0–0.7%). During 2015 to 2019, Guegan et al. [32] analyzed a total of 929 *A. fumigatus* isolates from patients with cystic fibrosis for azole resistance. The rate of ITC resistant *A. fumigatus* isolates was 14.5% (95/656); they were recovered from 44/308 (14.3%) patients. The median MIC of ITC-resistant isolates was 14 mg/L^−1^.

In a recently published study from Iran [33] MIC/MIC_50/90_ values of ITC were reported for *Aspergillus* section *Fumigati* and *Nigri* as 4/≥8 µg mL^−1^ (each) and 0.5/1 µg mL^−1^ for *Aspergillus* section *Terrei*. They also no NWT strains reported in 233 *Aspergillus* isolates collected from 11 university hospitals in Iran. In addition, in two other studies from Iran, none of the *A. terreus* isolates [34] and different species of aspergillus including *A. tubingensis* and *A. niger* [35] had a MIC of ≥ ECV for ITC. In contrast to these studies, Nargesi et al. [3] reported a rate of 7.3% of NWT strains *of A. niger* against ITC. They also reported MIC_50_/_90_ for *A. niger* and *A. tubingensis* against ITC as 0.25/0.5 and 0.5/16 µg mL^−1^, respectively. In this current study, all tested *Aspergillus* species represented MICs under ECV against VOR, ISV and POS but MIC > 16 to ITC. 

Despite all the measures that have been taken in the field of diagnosis of IA, the increasing prevalence of azole resistance has been found as a barrier in controlling this disease [36]. Small whole genome sequencing is a powerful technique which is used to capture variants throughout the entire genomic content of fungi and it provides a large amount of information in a short period of time compared to the approach target (such as Sanger sequencing) [37]. In azole-resistant strains of *Aspergillus* spp., the cyp51A and *cyp51B* genes may be exposed to polymorphism occurrence [13] The evidences gleaned from whole genome sequencing of *Aspergillus* paved the way for a clear description of how these genes operate without efforts to design multiple primers [14]. In regard to *A. fumigatus*, the majority of the existing data on tracking the effects of SNPs concentrate on defined sequences, such as the promoter region, TR34/L98H, of the *cyp51A* gene [38,39]. *A. niger* and *A. tubingensis* are two closely related species in the section *Nigri* [40]. The antifungal susceptibility profiles of these two species have already been reported in Iran [41] and in other countries [42]. *A. fumigatus* is more clear-cut in mechanisms of azole resistance than black *aspergilli.* Howard et al. [43] study on *cyp51A* gene mutations in multiple *A. niger* isolates showed contradictions in the concerned clades. They reported no mutation-related azole resistance in *cyp51A* gene in one clade, while six replacement mutations occurred in *A. niger* isolates of the other clade. Despite that, in our work, which overshadowed a larger scale of the genome, *A. niger*, with the exception of 15 silent mutations detected in the *cyp51A* and *cyp51B* genes, 3 and 2 replacement mutations were detected in *cyp51A* and *cyp51B* genes, respectively. Additionally, we detected 20 and 21 silent mutations in the *cyp51B* gene in *A. tubingensis* (isolates AT1 and AT2), respectively, despite the lack of mutations in the *cyp51B* gene (Table 2). *A. welwitschiae* is another member of the section *Nigri* [44]. Recent research showed that among the *A. niger* complex species, *A. welwitschiae* has a high isolation rate from clinical cases [39]. On the other hand, the antifungal susceptibility trend of *A. welwitschiae* has also been shown a higher MIC to VOR [45]. However, there is no exact information on how azole resistance has developed in *A. welwitschiae.* Based on the evidence from the whole genome sequencing of *A. welwitschiae*, substitutions at *cyp51A* and *cyp51B*, the two major target genes in *A. welwitschiae* isolate, as well as polymorphisms at multiple sites of target loci may explain the mechanism of ITC-resistance in our study. Zoran et al. [46] conducted a wide-range study on the azole resistance mechanism in *Aspergillus terreus.* Analysis of *cyp51A* gene, SNPs on 26 *A. terreus* isolates revealed that substitution at positions T650C, A649G, G1030A, and A956G resulted in changes in amino acid sequences. In another study performed by Rivero-Menendez et al. [47] on 12 triazole-resistant isolates of *A. terreus*, substitutions in D344N were perceived in two isolates and M217I in one isolate. In this study, between two isolates of *A. terreus*, only AR2 isolate showed a non-synonymous point mutation in the *cyp51A* and *cyp51B* genes separately (Table 2).

According to our findings, *Aspergillus* species isolated from different parts of the world may represent different pattern of resistant mechanisms including mutation in *cyp51A* and *cyp51B* genes which is clearly revealed by WGS. To show this, we made a literature review on the reported mutation in *Aspergillus fumigatus* revealed by Sanger sequencing of *cyp*51A and *cyp51B* genes (Table 3).

In this present study, we also evaluated non-*cyp51* gene loci for mutations to study *Aspergillus* isolates. This is the first report from Iran. According to our findings, many mutations were observed in different isolates. The most isolates showed simultaneously mutations in *cyp51* and non-*cyp51* gene loci. However, one isolate of *A. tubingensis* (AT1) and *A. terreus* (AR2) did not show *cyp51* mutations but AT2 and AR2 isolates showed mutations in *AtrR*, and *Cdr1B* and *AtrR*, respectively. *A. fumigatus* and AT1 (*A. tubingensis*) showed no mutations in *cyp51B.* However, they revealed mutations in non-*cyp51* genes including *AtrR* and *Hmg1*, and *Cdr1B*, *HapE* and *FfmA*, respectively. It is worth to note that mutations in *cyp51A* gene associated with azole resistance are well known. However, different studies have been recently reported that some other gene loci including ATP-Binding Cassette Transporters (*Cdr1B*)*, AtrR, Hmg1, HapE* and *FfmA* are required for wild-type azole resistance in *A. fumigatus* as the most studied species in *Aspergillus* genus [17,18,19,20,21]. Likewise, it was suggested that the most common azole resistance allele currently described is a linked change corresponding to a change in the coding sequence of *cyp51A* and a duplication of a 34-bp region in the promoter leading to a tandem repeat [40,48]. Paul et al. [18] also identified a positively acting transcription factor called *AtrR* that binds to the promoter of *cyp51A* as well as that of an important membrane transporter protein gene called *Cdr1B* (abcG1). These evidences have shown that non-*cyp51* gene loci such as *Cdr1B*, *AtrR*, *Hmg1*, *HapE* and *FfmA* are required for the synergistic increase in azole resistance and regulation of many different processes involved in drug resistance. Our finding on the homology modeling and mutation mappings of CYP51A of the selected *Aspergillus* species also suggest that the *cyp51A* may need other gene loci to cause azole resistance in *Aspergillus* species.

**Table 3:** An overview of the effects of point mutations on the emergence of azole resistance in *Aspergillus* spp. in the past decade.

*Aspergillus spp.*	Target Genes	Point Mutations	Mutation Results	Authors (Year) [Reference]
*A. fumigatus*	*Cyp51A*	TR34/L98H	Observation of ITC-Resistant isolates + Observation of VOR-resistant isolates	Camps et al. (2012) [49]
*A. fumigatus*	*Cyp51A*	TR34/L98H	Observation of Triazole-Resistant isolates	Chowdhary et al. (2012) [50]
*A. fumigatus*	*Cyp51A*	TR34/L98H TR46/Y121F/T289A	Observation of ITC-Resistant isolates Observation of VOR-Resistant isolates	Astvad et al. (2014) [51]
*A. fumigatus*	*Cyp51A*	TR34/L98H	Observation of ITC-Resistant isolates	Ahmad et al. (2014) [52]
*A. fumigatus*	*Cyp51A*	TR34/L98H TR46/Y121F/T289A	Observation of Triazole-Resistant isolates Observation of Triazole-Resistant isolates	van Ingen et al. (2015) [53]
*A. fumigatus*	*Cyp51A*	TR34/L98H F46Y, D255E, and M172- G54E	Observation of Triazole-Resistant isolates Observation of Triazole-Resistant isolates -High MICs to both ITC and POS	Chowdhary et al. (2015) [54]
*A. fumigatus*	*Cyp51A*	TR34/L98H	Observation of ITC-Resistant isolates + Observation of VOR-resistant isolates	Nabili et al. (2016) [55]
*A. fumigatus*	*Cyp51A*	TR34/L98H TR46/Y121F/T289A	Observation of ITC-Resistant isolates + Observation of VOR-resistant isolates	Wiederhold et al. (2016) [56]
*A. fumigatus*	*Cyp51A*	TR34/L98H	Observation of ITC-Resistant isolates + Observation of VOR-resistant isolates	Mohammadi et al. (2016) [57]
*A. fumigatus*	*Cyp51A*	TR34/L98H	Observation of ITC-Resistant isolates	Hurst et al. (2017) [58]
*A. fumigatus*	*Cyp51A*	TR34/L98H TR34/L98H/S297T/F495I G54V	Observation of ITC-Resistant isolates	Deng et al. (2017) [59]
*A. fumigatus*	*Cyp51A*	TR_34_/L98H/S297T/F495I	Observation of ITC-Resistant isolates	Chen et al. (2018) [60]
*A. fumigatus*	*Cyp51A*	TR34/L98H	Observation of ITC-Resistant isolates	Morio et al. (2018) [61]
*A. fumigatus*	*Cyp51A*	TR 34/L98H	Observation isolates with high MICs for ITC, VOR and POS	Paluch et al. (2019) [62]
*A. fumigatus*	*Cyp51A*	TR34/L98H G448S M220I	Observation of ITC-Resistant isolates	Tsuchido et al. (2019) [63]
*A. fumigatus*	*Cyp51A*	TR34/L98H/S297T/F495I	Observation isolates with high MICs for ITC, VOR and MCZ	Pontes et al. (2020) [64]
*A. fumigatus*	*Cyp51A*	TR34/L98H	Observation of VOR-Resistant isolates	Zhang et al. (2020) [65]
*A. fumigatus*	*Cyp51A*	TR34/L98H G448S	Observation of ITC-Resistant isolates Observation of VOR-resistant isolates	Gonzalez-Jimenez et al. (2021) [38]
*A. fumigatus*	*Cyp51B*	G457S	Observation of ITC-Resistant isolates + Observation of VOR-resistant isolates+ Observation of POS-resistant isolates+ Observation of ISV-resistant isolates	Gonzalez-Jimenez et al. (2020) [16]

## 5. Conclusions

Our study indicates that cryptic species not only have a spread in clinical cases but also a high proportion of point mutations leading to azole resistance in their genomic composition. There is a possibility to say that this volume of mutations did not occur all at once in cryptic species, but the techniques of identifying these species have entered a new era and with the correct identification of species, researchers have been able to observe and confront the mutations that occur continuously. Further studies on the effect of mutations in *cyp51* and non-*cyp51* gene loci to cause clinical resistance in *Aspergillus* isolates are warranted.

## Figures and Tables

**Table 1:** The clinical source and accession number of isolates.

Sample Code	Species	Section	Accession Number (*benA* Gene)	Origin
AF	*A. fumigatus*	*Fumigati*	MH208797	Patients with ABPA
AN	*A. niger*	*Nigri*	MH208734	Nail (Onychomycosis)
AW	*A. welwitschiae*	*Nigri*	MH208730	Sputum (asthmatic patient)
AT1	*A. tubingensis*	*Nigri*	MH208733	BAL (Patient with probable IA)
AT2	*A. tubingensis*	*Nigri*	MH208779	Nail (Onychomycosis)
AR1	*A. terreus*	*Terri*	MH208803	Sputum (asthmatic patient)
AR2	*A. terreus*	*Terri*	MH208818	BAL (Patient with probable IA)

BAL: Broncho alveolar lavage fluid, IA: invasive aspergillosis, ABPA: Allergic Bronchopulmonary aspergillosis.

## Data Availability

Not applicable.
